# Epidemiology, prevention, diagnosis, treatment, and outcomes for psychosocial problems in patients and families affected by non-intellectually impairing craniofacial malformation conditions: a systematic review protocol of qualitative data

**DOI:** 10.1186/s13643-019-1045-1

**Published:** 2019-05-27

**Authors:** Mikaela I. Poling, Craig R. Dufresne

**Affiliations:** Buckhannon, USA

**Keywords:** Craniofacial abnormalities, Body physical appearance, Craniofacial deformities, Reconstructive surgical procedures, Interpersonal relations, Self-concept, Social adjustment, Social support, Freeman-Sheldon syndrome, Whistling face syndrome

## Abstract

**Background:**

Physical attractiveness or unattractiveness wields a tremendous impact on the social and psychological components of life. Many individuals with facial deformities are treated more negatively than normal individuals, which may affect their self-image, quality of life, self-esteem, interpersonal encounters, and ultimately, success in life. Malformations that do not create physiological problems and whose major health impact is to degrade physical attractiveness and engender psychosocial consequences are insufficiently understood and not considered functional problems by medical insurance companies.

**Methods/design:**

As part of a clinical practice guideline development process for psychosocial concerns in Freeman-Burian syndrome, manuscripts describing psychosocial considerations related to the presence of non-intellectually impairing craniofacial malformation conditions or associated clinical activities are sought, especially focusing on epidemiology, prevention, symptoms, diagnoses, severity, timing, treatment, consequences, and outcomes. All published papers on this topic are considered in searching PubMed, OVID MEDLINE, and CINAHL Complete and again before final analyses. The results will be written descriptively to be practically useful and structured around the type or timing of psychosocial problems or consequences described or target population characteristics. No meta-analysis is planned.

**Discussion:**

Because the quality of research on psychosocial problems in craniofacial malformation conditions is known to be fraught with methodological problems, inconsistencies, and considerable knowledge gaps, we anticipate difficulties, which may limit the review questions able to be answered. We hope to produce a survey relevant to all non-intellectually impaired craniofacially deformed patients and their families and outline knowledge gaps and prioritise areas for clinical investigation.

**Systematic review registration: PROSPERO CRD42018093021:**

**Universal trial number: U1111-1211-8153:**

**Electronic supplementary material:**

The online version of this article (10.1186/s13643-019-1045-1) contains supplementary material, which is available to authorized users.

## Introduction

The experience of complex craniofacial syndromes frequently is a significant stressor for patients and families, and craniofacial malformations are the major contributor to this stress and negatively impact quality of life, mental health, socio-economic outcomes, long-term health, and health care use and costs [1–4]. Physical attractiveness or unattractiveness wields a tremendous impact on the social and psychological components of life [1]. Many individuals with facial deformities are treated more negatively than normal individuals, which may affect their self-image, quality of life, self-esteem, interpersonal encounters, and ultimately, success in life [1, 5]. Self-image is a major factor in creating a healthy self-esteem in the rehabilitation of craniofacially deformed patients [6]. Malformations that do not create physiological problems and whose major health impact is to degrade physical attractiveness and engender psychosocial consequences are insufficiently understood and not considered functional malformations [7]. Medical insurance companies commonly deny payment for reconstruction to aid in psychosocial healing for malformations that are psychologically but not physiologically functional [7]. While the background for this systematic review is not explicitly grounded in a specific level of evidence, the concepts asserted are well-known and widely acknowledged and investigated by many authors.

This systematic review protocol describes the process and methods by which manuscripts containing qualitative information on psychosocial considerations related to the presence of non-intellectually impairing craniofacial malformation conditions or associated clinical activities are sought. In addition to patient and family demographics, information on epidemiology, prevention, symptoms, diagnoses, severity, timing, treatment, consequences, and outcome of any psychosocial concerns for patients and their families are specifically sought. Primary outcome is the influence of craniofacial malformation conditions on the psychosocial health of patients and families, and the secondary outcome is the functional result (educational, occupational, quality of life, etc.) of the influence of craniofacial malformation conditions on the psychosocial health of patients and families. These outcomes do not assert whether craniofacial malformation conditions have a direct or indirect influence on psychosocial health; rather, the goal they address is to focus on any influence that may exist and its result, as demonstrated in the life of the patient and the patient’s family.

**Fig. 1:**
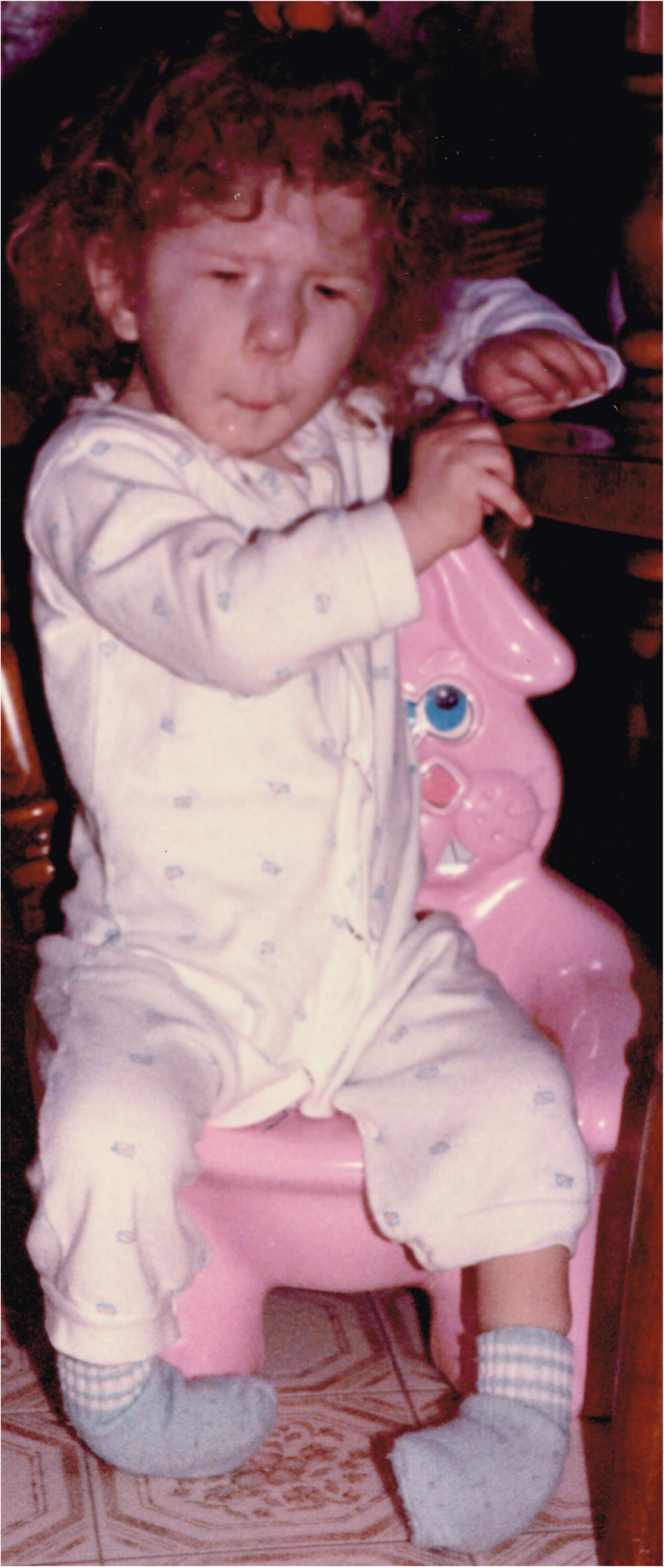
Child aged 1 year and 8 months with a typical presentation of Freeman-Burian syndrome (FBS). In addition to required features of microstomia, whistling-face appearance (pursed lips), H-shaped chin defect, prominent nasolabial folds, bilateral camptodactyly, ulnar deviation, metatarsus varus, and equinovarus, the patient exhibits numerous other craniofacial stigmata of FBS, including blepharophimosis and blepharoptosis, small nose, alar naris hypoplasia, lengthened philtrum, symmetrical midface hypoplasia, and micrognathia

This systematic review is expansion and update of an unstructured review previously published by the senior author (CRD) [1]. The systematic review will also serve as the underpinning for part of a comprehensive clinical practice guideline on Freeman-Burian syndrome (FBS; MIM 193700) (Fig. 1), formerly Freeman-Sheldon syndrome [8]. FBS is a rare congenital myopathic craniofacial syndrome [9], about which relatively little is known, and with the exception of our recently published anaesthesia recommendations [10], no clinical guidelines are available. Considerable clinical variability in severity is observed in FBS patients, but diagnosis requires the following: microstomia, whistling-face appearance (pursed lips), H- or V-shaped chin defect, and prominent nasolabial folds (Fig. 1) [11]. Some patients do not have limb malformations, but essentially all do, typically camptodactyly with ulnar deviation of the hand and talipes equinovarus (Fig. 1) [11]. Because no substantial scholarship exists on psychosocial influences in the lives of patients with FBS and their families [11], information from other craniofacial malformation disorders will serve to inform clinical decision-making in concert with the few case reports that mention psychosocial aspects of patient’s lives and our experience.

The protocol for a systematic review and meta-analysis specifically covering FBS has previously been separately registered (PROSPERO: CRD42015024740) and published [12], and a comprehensive unstructured narrative review based on that protocol has also been published [11]. Though most patients with craniofacial malformation conditions, including FBS, are diagnosed at birth or in early childhood, the goal is to produce a survey relevant to all patients with a suspected or confirmed FBS diagnosis and their families and to a lesser degree to all patients with non-intellectually impairing craniofacial malformation conditions and their families. While primarily directed toward psychiatrists, craniofacial surgeons, and teams caring for these patients and their families, the broader purpose of this protocol is to produce a survey of meaningful and clinically relevant contextual information for all who encounter these patients and interact with their families and to outline knowledge gaps and prioritise areas for clinical investigation.

## Methods and design

Undertaken as part of the unfunded FBS clinical practice guideline development process sponsored by Freeman-Sheldon Research Group, Inc., this systematic review protocol has received institutional review board (IRB) approval from FSRG IRB #1 and is registered with the World Health Organization (U1111-1211-8153) and on PROSPERO (CRD42018093021), where subsequent amendments are available. Neither the sponsor nor any institution has a role in protocol development or associated activities. The protocol, including development of actionable clinical questions (Table 1), has been prospectively drafted in compliance with Preferred Reporting Items for Systematic Reviews and Meta-Analyses Protocols [13] (Additional file 1: PRISMA-P 2015 Checklist).

**Table 1:** Actionable guiding clinical questions

1.	For patients of any age with non-intellectually impairing craniofacial malformation conditions, are the social consequences of facial deformities or their own affective reaction to the appearance abnormality more troubling in most of their social settings (e.g. school, work, leisure activities enjoyed with others or in public spaces)?
2.	For patients of any age with non-intellectually impairing craniofacial malformation conditions, do non-facial aesthetic psychosocial confounds nullify the facial deformity’s effect contribution to the psychosocial burden in most of their social settings (e.g. school, work, leisure activities enjoyed with others or in public spaces)?
3.	Compared with healthy individuals raised as children in the home, does the psychosocial burden for patients of any age with non-intellectually impairing craniofacial malformation conditions raised as children in the home involve manifestation of significant psychopathology?
4.	Compared with healthy adults raised as children in the home, does the psychosocial burden for patients of any age with non-intellectually impairing craniofacial malformation conditions raised as children in the home involve manifestation of significant impairment into adulthood?
5.	For school age children with non-intellectually impairing craniofacial malformation conditions, do parents and teachers rate psychosocial competency more differently than expected for healthy children observed in similar social settings (e.g. school, family time, leisure activities enjoyed with others or in public spaces)?
6.	Compared with healthy individuals raised as children in the home, is self-concept, self-image, or body image impaired for patients of any age with non-intellectually impairing craniofacial malformation conditions raised as children in the home?
7.	Compared with healthy individuals, do gender and other socio-demographic differences in patients of any age with non-intellectually impairing craniofacial malformation conditions and those who interact with these patients (e.g. parents, teachers, raters in experimental studies) modify effect of the patient’s facial deformity on psychosocial reactions in most of social settings (e.g. home, family activities, school, work, leisure activities enjoyed with others or in public spaces)?
8.	Compared with healthy individuals raised as children in the home, do patients of any age with non-intellectually impairing craniofacial malformation conditions and their families identify more or fewer positive psychosocial experiences?
9.	For patients of any age with non-intellectually impairing craniofacial malformation conditions, does misinformation or a lack of appropriate and timely information conveyed during outpatient and inpatient medical encounters by various providers (e.g. geneticists, paediatricians, nurses, and others) constitute a psychosocially important secondary trauma or inconvenience?
10.	For patients of any age with non-intellectually impairing craniofacial malformation conditions, does folklore or other preconceived ideas about ‘crippled children’ or objective knowledge about causes and treatment of non-intellectually impairing craniofacial malformation conditions have a larger influence in the psychosocial atmosphere of the immediate and extended family at home and during family social activities?
11.	For infants with non-intellectually impairing craniofacial malformation conditions, is maternal attachment or interaction impaired in most of settings (e.g. home, daycare, activities with others or in public spaces, medical encounters) compared to that observed in healthy infants?
12.	For patients of any age with non-intellectually impairing craniofacial malformation conditions compared with healthy individuals, does the experience of social or occupational discrimination lie outside of the range expected in similar social settings (e.g. school, family time, leisure activities enjoyed with others or in public spaces)?
13.	For patients of any age with non-intellectually impairing craniofacial malformation conditions compared with healthy individuals in similar social settings (e.g. school, family time, leisure activities enjoyed with others or in public spaces), does a real or perceived social or occupational discrimination present a functional or perceived burden, psychosocial deterrent, or inconvenience?
14.	Compared to a healthy child’s birth and under similar psychosocial circumstances and healthcare setting and who is then raised in the home, do families identify the birth of a child with a non-intellectually impairing craniofacial malformation condition primarily as a psychosocially important trauma, burden, or joy?
15.	Compared to raising a healthy child at home, do families identify raising a child at home with a non-intellectually impairing craniofacial malformation condition primarily as a psychosocially important burden, logistical burden, or other type of dramatic experience?
16.	For patients of any age with non-intellectually impairing craniofacial malformation conditions and their families, are there key psychological needs that should be addressed within the setting of the craniofacial team or is a general conversation about typical psychosocial concerns within this population sufficient to reduce psychosocial morbidity for patients and their families?
17.	Compared with parents of healthy children raised in the home, what psychosocial support, assessment, and care do parents of children with non-intellectually impairing craniofacial malformation conditions require to minimize stress-related problems and maintain the integrity of the family and self-image of the patient?
18.	Compared with healthy children raised in the home, what psychosocial support, assessment, and care do children with non-intellectually impairing craniofacial malformation conditions require to minimize stress-related problems and maintain the integrity of the family and self-image of the patient?
19.	Compared to the role of the primary care provider in the psychosocial management of healthy children raised in the home and their families, what is the role of the craniofacial surgeon in the psychosocial management of patients with non-intellectually impairing craniofacial malformation conditions raised in the home and their families to best minimize stress-related problems and maintain the integrity of the family and self-image of the patient?
20.	Compared with healthy children raised in the home, do patients with non-intellectually impairing craniofacial malformation conditions and their families more often require long-term psychosocial support to minimize stress-related problems and maintain the integrity of the family and self-image of the patient?

### Responsibility for execution of review stages

Unless otherwise stated, all stages of the systematic review (e.g. literature searches, data abstraction, pilot-testing of stages, screening of citations and full-text articles, assessment of risk of bias) are primarily executed by the junior author (MIP) and reviewed twice by the senior author (CRD). To ensure proper checks-and-balances of this process, the senior author repeats part or all of any stage of the systematic review at his discretion to validate the initial work of the junior author. Discrepancies and disagreements are resolved through discussion between the authors or through consultation with the ethics director or his designee. All procedures are unblinded.

### Literature search

Material for consideration is identified by searching PubMed database on 26 September 2017 and both OVID MEDLINE and CINAHL Complete on 20 April 2019 for all articles in any language relating to psychosocial difficulties in non-intellectually impairing craniofacial malformation conditions. No advanced search features or limits are used because of the paucity of available literature. The PubMed search strategy (https://www.crd.york.ac.uk/PROSPEROFILES/93021_STRATEGY_20180408.pdf) comprises relevant Medical Subject Headings vocabulary as search terms, including as follows: reconstructive surgical procedures/psychology, craniofacial deformities, craniofacial abnormalities/psychology. Additionally, the Links from PubMed feature is used to retrieve results similar to one article (PMID: 21440966). Through preliminary testing of different selections of terms and conditions, we have developed a list of terms that produced the best balance between comprehensiveness and relevance, with an exhaustive list of craniofacial malformation conditions being judged as too broad and not offering any improvement in catchment of descriptions of psychosocial problems. The junior author will primarily conduct the searches, as the junior author was trained by an experienced medical librarian to execute these types of searches and how best to employ various databases. Searches are re-run before final analyses, and articles published since the initial search are retrieved for inclusion. Appropriate citations from the original unstructured review are used [1]. Finally, searches are peer reviewed using the Peer Review of Electronic Search Strategies (PRESS) Checklist [14, 15] by librarians experienced conducting these queries.

### Eligibility criteria

Manuscripts primarily discussing psychosocial considerations in craniofacially disfigured patients without intellectual impairment are considered for inclusion. Exclusion criteria comprise any manuscripts primarily discussing (1) psychosocial considerations in patients exhibiting non-pathological variation in dentofacial anatomic relationships (such as need or desire for dental braces or other orthognathic treatment not associated with an underlying malformation syndrome or condition), (2) craniofacially disfigured patients with intellectual impairment, (3) craniofacially disfigured patients without intellectual impairment but not addressing psychosocial considerations to any significant extent, or (4) psychosocial considerations in non-craniofacially disfigured patients. Treatment comparison is expected to be limited, as many articles describe neither interventions nor outcomes; those that do cannot be confidently compared, due to high inter-patient and inter-intervention variability. It is not certain that many of the guiding questions are able to be answered, and the objectives more clearly reflect expectations for what data will be available. No published articles are excluded based on design, but book chapters and books are excluded from consideration. Most non-English language articles are translated (Russian, Czech, German) in-house. Asian language articles are unable to be reviewed or translated in-house and are excluded. There are no restrictions on patient gender, ethnicity, geographical location, religion, socio-economic status, or clinical setting.

### Data extraction

As searches using the search strategy and examination of the original unstructured review [1] are carried out, citations and abstracts are placed in a single word processor document file for screening to identify those potentially meeting the inclusion criteria. After screening, abstracts of articles are assessed for eligibility before inclusion. Any citation not having an abstract is excluded at this point. After eligibility assessment of abstracts and elimination of duplicates, narrative summaries containing prescribed data sought (Table 2) are drafted for each citation and placed in a single word processor document file. Selected summaries that best reflect the goals of the systematic review are then incorporated into the manuscript. Authors of articles being considered are not contacted with data queries. Final selection is based on subjective assessment of similarity to experiences encountered by patients with FBS and their families. Based on our empirical understanding, the experience of FBS for patients and families is typified by a collection of frustrating and emotionally draining, physically exhausting, and potentially traumatic situations. FBS patients require resource-intensive care in infancy and childhood and have many medical encounters with various specialities. Because of the rarity of the syndrome, families often receive conflicting or false information from the medical community. Patients undergo multiple surgeries and face lifelong syndrome-related physical challenges and social stigma.

**Table 2:** Qualitative data and outcomes sought in manuscripts describing psychosocial problems in patients and families affected by non-intellectually impairing craniofacial malformation conditions. Some qualitative data items and outcomes are entirely or mostly subjective and pose a substantial risk for bias

Minimum data extracted:	
Patient population	
Psychosocial problem or diagnosis	
Other data sought:	
Clinical and functional outcome	
Patient, parental, and others’ attitudes toward craniofacial malformations	
Psychosocial effect on family functioning and marital integrity	
Parental and others’ reaction to the birth of a craniofacially deformed child	
Effect of misinformation on parents	
Impact of craniofacial malformations on development of self-concept, self-image, and body image	
Role of gender and other socio-economic and demographic confounds in patient, parental, and others’ attitudes toward the craniofacially deformed	
Positive aspects in the experience of a craniofacial malformation condition	
Congruence of teachers’, parents’, and patients’ opinions on the psychosocial development of craniofacially deformed children	
Contribution of aesthetic facial appearance versus non-aesthetic features to patient, parental, and others’ attitudes toward the craniofacially deformed	

### Risk of bias (quality) assessment

Because of the aforementioned methodological limitations in this area of literature, bias risk assessment may be limited. ROBIS [16] will be used for bias assessment. In the context of this systematic review, bias assessment must also account for the review authors’ judgement of the article’s clinical relevance for patients with Freeman-Burian syndrome. Considerable bias is expected and accepted as unavoidable. Grading of Recommendations Assessment, Development and Evaluation [17] will be used for evidence.

### Strategy for data synthesis and analysis of subgroups or subsets

The results will be written descriptively to be practically useful and structured around the type or timing of psychosocial problems or consequences described or target population characteristics (Table 3). In an exploratory, informal literature survey conducted to develop this protocol, we have found that many authors referred to a great diversity of methodological problems in this area of the literature. Therefore, no meta-analysis, subgroup analyses, or formal assessment of the strength of the body of evidence are planned due to anticipated infeasibility, based on these previously described methodological problems in this area of the literature. The final systematic review manuscript draft will be internally peer reviewed by at least two colleagues in psychiatry; however, use of a structured instrument for the internal peer review is not planned.

**Table 3:** Findings from the included manuscripts, structured around the type or timing of the psychosocial problem or consequences described or target population characteristics

1. Psychosocial considerations reported in Freeman-Burian syndrome	
2. Facial aesthetics	
3. Non-facial aesthetic psychosocial confounds	
4. Psychosocial considerations reported in other craniofacially deformed patients	
5. Parental perspective on children’s psychosocial status	
6. Impact on self-concept, self-image, and body image	
7. Gender differences in psychosocial reactions	
8. Positive aspects of the experience of a craniofacial condition	
9. Trauma of misinformation	
10. Attitudes toward the craniofacially deformed	
11. Tragedy of the birth of a craniofacially deformed child	
12. Parental and family psychosocial burden of a craniofacially deformed child	
13. Attitudes of patients with Freeman-Burian syndrome	
14. Psychiatric assessment and continued support	

## Discussion

The quality of research on psychosocial problems in craniofacial malformation conditions is known to be fraught with methodological problems, inconsistencies, and considerable knowledge gaps [18–29]. Much of the literature on the psychosocial impact of craniofacial deformities focuses on cleft lip and palate [21]. While there are undoubtedly psychosocial and therapeutic similarities for cleft lip and palate and other more extensive malformation conditions from which inferences can be drawn, each craniofacial condition is unique and likely to have distinct psychosocial consequences [30]. In cleft lip and palate patients, the tissue is discontinuous but histologically normal and reacts appropriately to operative repair. In other more extensive malformation conditions, such as FBS, the tissue and biochemistry may be atypical and poorly understood. This pathological difference can herald a worse psychosocial course than suggested by the literature describing patients with cleft lip and palate and other deformational type diagnoses.

## Additional file

Additional file 1: PRISMA-P 2015 Checklist (PDF 104 kb)

## Data Availability

Upon completion of the systematic review, manuscripts describing the results and their implications will be submitted to appropriate peer-reviewed journals in craniofacial surgery and rare diseases. Any resulting published manuscripts (or a summary thereof) and supplemental data will also be made available on the review authors’ ResearchGate.net profile pages.

## Abbreviations

FBS: Freeman-Burian syndrome
IRB: Institutional review board
MIM: Mendelian Inheritance in Man
