# Supplementary material for: Epidemiology, prevention, diagnosis, treatment, and outcomes for psychosocial problems in patients and families affected by non-intellectually impairing craniofacial malformation conditions: a systematic review protocol of qualitative data
Source: Syst Rev. 2019 May 27;8:127. doi: 10.1186/s13643-019-1045-1 (PMC6537209; doi:10.1186/s13643-019-1045-1)
Supplement: Supplementary file 1 — PRISMA-P 2015 Checklist (PDF 104 kb) [file 13643_2019_1045_MOESM1_ESM.pdf]

PRISMA-P 2015 Checklist

This checklist has been adapted for use with systematic review protocol submissions to BioMed Central journals from Table 3 in Moher D et al: Preferred reporting items for systematic review and meta-analysis protocols (PRISMA-P) 2015 statement. *Systematic Reviews* 2015 **4**:1

An Editorial from the Editors-in-Chief of *Systematic Reviews* details why this checklist was adapted - **Moher D, Stewart L & Shekelle P:** Implementing PRISMA-P: recommendations for prospective authors. *Systematic Reviews* 2016 **5**:15

Section/topic	#	Checklist item	Information reported		Line number(s)
			Yes	No	
**ADMINISTRATIVE INFORMATION**					
**Title**					
Identification	1a	Identify the report as a protocol of a systematic review	☒	☐	P 1: Line 14
Update	1b	If the protocol is for an update of a previous systematic review, identify as such	☒	☐	NA
**Registration**	2	If registered, provide the name of the registry (e.g., PROSPERO) and registration number in the Abstract	☒	☐	P 2: Line 54, 56
**Authors**					

Section/topic	#	Checklist item	Information reported		Line number(s)
			Yes	No	
Contact	3a	Provide name, institutional affiliation, and e-mail address of all protocol authors; provide physical mailing address of corresponding author	☒	☐	P 1: Line 21-23, 27-29; P 11: Line 45-52
Contributions	3b	Describe contributions of protocol authors and identify the guarantor of the review	☒	☐	P 11: Line 12, 30-32
**Amendments**	4	If the protocol represents an amendment of a previously completed or published protocol, identify as such and list changes; otherwise, state plan for documenting important protocol amendments	☒	☐	P 5: Line 12-14; P 6: Line 19-21
**Support**					
Sources	5a	Indicate sources of financial or other support for the review	☒	☐	P 11: Line 14; P 6: Line 12
Sponsor	5b	Provide name for the review funder and/or sponsor	☒	☐	P 11: Line 16-18; P 6: Line 14
Role of	5c	Describe roles of funder(s), sponsor(s), and/or institution(s), if any, in developing the protocol	☒	☐	P 11: Line 16-

Section/topic	#	Checklist item	Information reported Yes	Information reported No	Line number(s)
sponsor/funder					18; P 6: Line 21-23
**INTRODUCTION**					
**Rationale**	6	Describe the rationale for the review in the context of what is already known	☒	☐	P 2: Line 7-21; P 4: Line 7-39; P 5: Line 16-39, 48-54; P 8: Line 43-56; P 9: Line 43-59; P 10: Line 5-10
**Objectives**	7	Provide an explicit statement of the question(s) the review will address with reference to participants, interventions, comparators, and outcomes (PICO)	☒	☐	Table 1
**METHODS**					
**Eligibility criteria**	8	Specify the study characteristics (e.g., PICO, study design, setting, time frame) and report characteristics (e.g., years considered, language, publication status) to be used as criteria for eligibility for the review	☒	☐	P 2: Line 23-32; P 7: 39-59; P 8: Line 5-19;

Section/topic	#	Checklist item	Information reported		Line number(s)
			Yes	No	
					Table 1
**Information sources**	9	Describe all intended information sources (e.g., electronic databases, contact with study authors, trial registers, or other grey literature sources) with planned dates of coverage	☒	☐	P 2: Line 34; P 6: Line 50-59; P 7: 5-34
**Search strategy**	10	Present draft of search strategy to be used for at least one electronic database, including planned limits, such that it could be repeated	☒	☐	P 7: Line 5
STUDY RECORDS					
Data management	11a	Describe the mechanism(s) that will be used to manage records and data throughout the review	☒	☐	P 8: 23-27, 32-39
Selection process	11b	State the process that will be used for selecting studies (e.g., two independent reviewers) through each phase of the review (i.e., screening, eligibility, and inclusion in meta-analysis)	☒	☐	P 6: Line 32-45; P 7: 21-25, 30-34
Data collection process	11c	Describe planned method of extracting data from reports (e.g., piloting forms, done independently, in duplicate), any processes for obtaining and confirming data from investigators	☒	☐	P 8: 23-43

Section/topic	#	Checklist item	Information reported		Line number(s)
			Yes	No	
**Data items**	12	List and define all variables for which data will be sought (e.g., PICO items, funding sources), any pre-planned data assumptions and simplifications	☒	☐	Table 2
**Outcomes and prioritization**	13	List and define all outcomes for which data will be sought, including prioritization of main and additional outcomes, with rationale	☒	☐	Table 2
**Risk of bias in individual studies**	14	Describe anticipated methods for assessing risk of bias of individual studies, including whether this will be done at the outcome or study level, or both; state how this information will be used in data synthesis	☒	☐	P 9: Line 7
DATA					
**Synthesis**	15a	Describe criteria under which study data will be quantitatively synthesized	☐	☒	NA
	15b	If data are appropriate for quantitative synthesis, describe planned summary measures, methods of handling data, and methods of combining data from studies, including any planned exploration of consistency (e.g., I^2, Kendall's tau)	☐	☒	NA
	15c	Describe any proposed additional analyses (e.g., sensitivity or subgroup analyses, meta-regression)	☐	☒	NA

Section/topic	#	Checklist item	Information reported		Line number(s)
			Yes	No	
	15d	If quantitative synthesis is not appropriate, describe the type of summary planned	☒	☐	P 2: Line 34-39; P 9: Line 21-25; Table 3
**Meta-bias(es)**	16	Specify any planned assessment of meta-bias(es) (e.g., publication bias across studies, selective reporting within studies)	☒	☐	P 9: Line 7
**Confidence in cumulative evidence**	17	Describe how the strength of the body of evidence will be assessed (e.g., GRADE)	☒	☐	P 9: Line 14-16

BioMed Central
The Open Access Publisher
